# Underlying Medical Conditions and Severe Illness Among 540,667 Adults Hospitalized With COVID-19, March 2020–March 2021

**DOI:** 10.5888/pcd18.210123

**Published:** 2021-07-01

**Authors:** Lyudmyla Kompaniyets, Audrey F. Pennington, Alyson B. Goodman, Hannah G. Rosenblum, Brook Belay, Jean Y. Ko, Jennifer R. Chevinsky, Lyna Z. Schieber, April D. Summers, Amy M. Lavery, Leigh Ellyn Preston, Melissa L. Danielson, Zhaohui Cui, Gonza Namulanda, Hussain Yusuf, William R. Mac Kenzie, Karen K. Wong, James Baggs, Tegan K. Boehmer, Adi V. Gundlapalli

**Affiliations:** 1 COVID-19 Response, Centers for Disease Control and Prevention, Atlanta, Georgia; 2 US Public Health Service Commissioned Corps, Rockville, Maryland; 3 Epidemic Intelligence Service, Center for Surveillance, Epidemiology, and Laboratory Services, Centers for Disease Control and Prevention, Atlanta, Georgia

## Abstract

**Introduction:**

Severe COVID-19 illness in adults has been linked to underlying medical conditions. This study identified frequent underlying conditions and their attributable risk of severe COVID-19 illness.

**Methods:**

We used data from more than 800 US hospitals in the Premier Healthcare Database Special COVID-19 Release (PHD-SR) to describe hospitalized patients aged 18 years or older with COVID-19 from March 2020 through March 2021. We used multivariable generalized linear models to estimate adjusted risk of intensive care unit admission, invasive mechanical ventilation, and death associated with frequent conditions and total number of conditions.

**Results:**

Among 4,899,447 hospitalized adults in PHD-SR, 540,667 (11.0%) were patients with COVID-19, of whom 94.9% had at least 1 underlying medical condition. Essential hypertension (50.4%), disorders of lipid metabolism (49.4%), and obesity (33.0%) were the most common. The strongest risk factors for death were obesity (adjusted risk ratio [aRR] = 1.30; 95% CI, 1.27–1.33), anxiety and fear-related disorders (aRR = 1.28; 95% CI, 1.25–1.31), and diabetes with complication (aRR = 1.26; 95% CI, 1.24–1.28), as well as the total number of conditions, with aRRs of death ranging from 1.53 (95% CI, 1.41–1.67) for patients with 1 condition to 3.82 (95% CI, 3.45–4.23) for patients with more than 10 conditions (compared with patients with no conditions).

**Conclusion:**

Certain underlying conditions and the number of conditions were associated with severe COVID-19 illness. Hypertension and disorders of lipid metabolism were the most frequent, whereas obesity, diabetes with complication, and anxiety disorders were the strongest risk factors for severe COVID-19 illness. Careful evaluation and management of underlying conditions among patients with COVID-19 can help stratify risk for severe illness.

**Summary**

**What is already known about this topic?**

Severe COVID-19 illness in adults has been linked to underlying medical conditions.

**What is added by this report?**

In this cross-sectional study of 540,667 adult hospitalized patients with COVID-19, 94.9% had at least 1 underlying medical condition. Hypertension and disorders of lipid metabolism were the most frequent, whereas obesity, diabetes with complication, anxiety disorders, and the total number of conditions were the strongest risk factors for severe COVID-19 illness.

**What are the implications for public health practice?**

Preventing COVID-19 in populations with these underlying conditions and multiple conditions should remain a public health priority, with targeted mitigation efforts and ensuring high uptake of vaccine, when available, in these individuals and their close contacts.

## Introduction

As the COVID-19 pandemic continues, a need remains to understand indicators for severe illness, defined as admission to an intensive care unit (ICU) or stepdown unit, invasive mechanical ventilation (IMV), or death (1). Several underlying medical conditions among adults, including diabetes, obesity, chronic kidney disease (CKD), hypertension, and immunosuppression, have been reported to be associated with increased risk for severe illness from COVID-19 (2-4). However, many existing studies are limited in geographic representation, restricted to cases early in the outbreak, or focused on a limited number of preselected conditions and/or severe outcomes (3–5). Finally, few studies have shown the effect of the number of underlying medical conditions on the risk for severe COVID-19 illness (6).

Both the baseline prevalence of a condition and the magnitude of its association with COVID-19 illness help determine the impact of a condition at a population level. This study, based on a large electronic administrative discharge data set, sought to describe the most frequent underlying medical conditions among hospitalized patients with COVID-19 and their associations with severe illness. This information can better inform clinical practice and public health priorities, such as identifying populations for focused prevention efforts and potential vaccine prioritization.

## Methods

We used the Premier Healthcare Database Special COVID-19 Release (PHD-SR, release date May 11, 2021), a large, US hospital-based, all-payer database (7). The sample included patients aged 18 years or older who had an inpatient encounter with an *International* *Classification of Diseases, Tenth Revision, Clinical Modification* (ICD-10-CM) diagnosis of U07.1 (“COVID-19, virus identified”) from April 1, 2020, through March 31, 2021, or B97.29 (“other coronavirus as the cause of diseases classified elsewhere,” recommended before the April 2020 release of U07.1) from March 1, 2020, through April 30, 2020 (8,9).

We examined 3 indicators of severe COVID-19 illness: admission to an ICU or stepdown unit, IMV, and death. These indicators were not mutually exclusive.

We considered 2 exposures of interest: 1) specific underlying medical conditions and 2) the number of conditions. We captured data on both exposures by using ICD-10-CM diagnosis codes from inpatient or outpatient hospital records in PHD-SR from January 2019 up to and including a patient’s first inpatient encounter for COVID-19. We used 1 encounter with an ICD-10-CM code to establish the presence of an underlying condition because few patients had multiple encounters in this hospital database. We excluded 3 ICD-10-CM codes (ie, oxygen support, dependence on a ventilator, and tracheostomy) listed during the patient’s COVID-19 encounter because they could be part of COVID-19 treatment.

We used a multistep approach to identify underlying medical conditions. First, we used the Chronic Condition Indicator (CCI) to identify chronic ICD-10-CM codes (11,803 of 73,205 total ICD-10-CM codes), which were then aggregated into 314 categories using the Clinical Classifications Software Refined (CCSR) (10,11). To further differentiate underlying conditions from acute complications of COVID-19, a panel of physicians (K.K.W., W.M.K., H.G.R., B.B., N.T.A., J.M.N.) classified the 314 CCSR categories into “likely underlying” (274 categories; eg, asthma); “indeterminate,” which could include underlying or acute complications or both (29 categories; eg, cardiac dysrhythmias); or “likely acute” (11 categories; eg, acute pulmonary embolism). We used the “likely underlying” CCSR categories for our analysis of underlying medical conditions and excluded the “indeterminate” or “likely acute” CCSR categories. People diagnosed with both CCSR categories of “diabetes with complication” and “diabetes without complication” (n = 55,141) were classified as having diabetes with complication. The number of underlying medical conditions was defined as the number of unique CCSR categories associated with each patient (0, 1, 2–5, 6–10, >10).

### Statistical analyses

We described the sample by patient and hospital characteristics. Then we selected the most frequent underlying CCSR categories with a prevalence of 10% or more in the sample. We used multivariable generalized linear models with Poisson distribution and log link function to estimate adjusted risk ratios (aRRs) for 3 outcomes of interest among hospitalized patients: ICU admission, IMV, and death (reference was surviving hospitalized patients without that outcome). We performed these estimations by 1) including all frequent CCSR categories in the same model (“full model”) and 2) including 1 CCSR category per statistical model (“restricted model”). We focused our interpretations on the CCSR categories whose direction of association (positive or negative) was consistent between the restricted and the full model. We also conducted a stratified analysis of frequent conditions by age group (frequency ≥10.0% in each age group). Finally, we estimated the association between the number of CCSR categories and the 3 severity outcomes.

All models used robust SEs clustered on hospital identification, and controlled for patient age, sex, race/ethnicity, payer type, hospital urbanicity, US Census region of hospital, admission month, and admission month squared (to account for potential nonlinear unobservable changes in treatment, patient profile, or severity of illness during the pandemic). All analyses were conducted using R version 4.0.2 (The R Foundation) and Stata version 15.1 (StataCorp LLC).

We performed 2 sensitivity analyses using all chronic CCSR categories, including those determined by the clinician panel to be “likely underlying,” “indeterminate,” and “likely acute.” We performed 1 sensitivity analysis in the main sample and another that was limited to encounters that preceded the first COVID-19 inpatient encounter. These analyses were used to validate the associations found in the main analysis, as well as to examine the conditions excluded from the main analysis after clinical review.

This activity was reviewed by the Centers for Disease Control and Prevention (CDC) and was conducted according to applicable federal law and CDC policy.

## Results

Among 4,899,447 hospitalized patients in PHD-SR, 540,667 (11.0%) patients met the study inclusion criteria for COVID-19 (Table 1). Of patients hospitalized with COVID-19, 94.9% had at least 1 documented underlying CCSR condition, 249,522 (46.2%) had an ICU admission, 76,680 (14.2%) received IMV, and 80,174 (14.8%) died. The study sample included 261,078 (48.3%) female patients, 94,670 (17.5%) non-Hispanic Black patients, and 93,171 (17.2%) Hispanic or Latino patients. The median age was 66 years, and the most common insurance types were Medicare (292,978 [54.2%]) and commercial (130,995 [24.2%]). The 863 hospitals visited by patients included in the study were distributed across all US Census regions.

**Table 1:** Characteristics of Adults Hospitalized With COVID-19 in Premier Healthcare Database Special COVID-19 Release (PHD-SR), March 2020–March 2021

Characteristic^a^	All Hospitalized Patients in PHD-SR, No. (%)	Hospitalized Patients With COVID-19, No. (%)			
		Full Sample	ICU^b^ admission	IMV^b^	Died^b^
**Total**	4,899,447 (100.0)	540,667 (100.0)	249,522 (100.0)	76,680 (100.0)	80,174 (100.0)
**No. of conditions**					
≥1^c^	4,438,183 (90.6)	513,292 (94.9)	242,372 (97.1)	75,514 (98.5)	79,434 (99.1)
0	461,264 (9.4)	27,375 (5.1)	7,150 (2.9)	1,166 (1.5)	740 (0.9)
1	402,499 (8.2)	39,776 (7.4)	14,272 (5.7)	2,785 (3.6)	2,087 (2.6)
2–5	1,796,770 (36.7)	212,429 (39.3)	94,405 (37.8)	27,405 (35.7)	25,893 (32.3)
6–10	1,565,845 (32.0)	167,706 (31.0)	84,745 (34.0)	28,774 (37.5)	31,310 (39.1)
>10	673,069 (13.7)	93,381 (17.3)	48,950 (19.6)	16,550 (21.6)	20,144 (25.1)
**Sex**					
Female	2,860,589 (58.4)	261,078 (48.3)	110,017 (44.1)	30,062 (39.2)	32,939 (41.1)
Male	2,037,012 (41.6)	279,317 (51.7)	139,416 (55.9)	46,587 (60.8)	47,211 (58.9)
Unknown	1,846 (0.0)	272 (0.1)	89 (0.0)	31 (0.0)	24 (0.0)
**Age, y**					
Median (IQR), y	68 (57–78)	66 (53–77)	67 (55–77)	67 (57–75)	74 (65–83)
18–39	1,304,324 (26.6)	59,697 (11.0)	19,120 (7.7)	4,192 (5.5)	1,299 (1.6)
40–49	428,000 (8.7)	51,591 (9.5)	22,605 (9.1)	5,913 (7.7)	2,710 (3.4)
50–64	1,085,170 (22.1)	144,306 (26.7)	68,791 (27.6)	22,791 (29.7)	14,867 (18.5)
65–74	923,004 (18.8)	121,832 (22.5)	62,056 (24.9)	23,055 (30.1)	21,421 (26.7)
75–84	735,429 (15.0)	103,012 (19.1)	50,891 (20.4)	16,041 (20.9)	23,308 (29.1)
≥85	423,520 (8.6)	60,229 (11.1)	26,059 (10.4)	4,688 (6.1)	16,569 (20.7)
**Race/ethnicity**					
Hispanic or Latino	530,274 (10.8)	93,171 (17.2)	44,423 (17.8)	13,826 (18.0)	12,319 (15.4)
Non-Hispanic White	3,199,707 (65.3)	293,558 (54.3)	136,577 (54.7)	38,969 (50.8)	45,738 (57.0)
Non-Hispanic Black	695,818 (14.2)	94,670 (17.5)	42,624 (17.1)	13,584 (17.7)	12,413 (15.5)
Non-Hispanic Asian	113,914 (2.3)	13,048 (2.4)	5,566 (2.2)	2,064 (2.7)	1,918 (2.4)
Other	254,042 (5.2)	33,155 (6.1)	14,530 (5.8)	5,898 (7.7)	5,620 (7.0)
Unknown	105,692 (2.2)	13,065 (2.4)	5,802 (2.3)	2,339 (3.1)	2,166 (2.7)
**Payer type**					
Commercial	1,331,431 (27.2)	130,995 (24.2)	56,625 (22.7)	15,844 (20.7)	9,641 (12.0)
Medicare	2,222,845 (45.4)	292,978 (54.2)	143,130 (57.4)	44,822 (58.5)	60,017 (74.9)
Medicaid	929,286 (19.0)	72,953 (13.5)	29,552 (11.8)	10,191 (13.3)	6,450 (8.0)
Charity/indigent/self-pay	211,569 (4.3)	16,644 (3.1)	7,304 (2.9)	2,041 (2.7)	1,267 (1.6)
Unknown	204,316 (4.2)	27,097 (5.0)	12,911 (5.2)	3,782 (4.9)	2,799 (3.5)
**Hospital US Census region**					
Midwest	1,098,092 (22.4)	111,532 (20.6)	49,080 (19.7)	15,878 (20.7)	15,680 (19.6)
Northeast	798,013 (16.3)	101,396 (18.8)	33,702 (13.5)	14,273 (18.6)	17,749 (22.1)
South	2,261,510 (46.2)	251,627 (46.5)	123,465 (49.5)	34,647 (45.2)	35,276 (44.0)
West	741,832 (15.1)	76,112 (14.1)	43,275 (17.3)	11,882 (15.5)	11,469 (14.3)

Abbreviation: IQR, interquartile range.

a Some categories may not add up to 100% because of rounding.

b Columns are not mutually exclusive.

c Underlying medical conditions were defined by 1) using Chronic Condition Indicator to identify chronic *International Classification of Diseases, Tenth Revision, Clinical Modification* codes; 2) aggregating the codes into a smaller number of meaningful categories using the Clinical Classifications Software Refined (CCSR); 3) a clinical review of CCSR categories that classified the CCSR codes as “likely underlying,” “indeterminate,” and “likely acute”; and 4) including only “likely underlying” CCSR categories and excluding “indeterminate” and “likely acute” CCSR categories.

**Figure 1:**
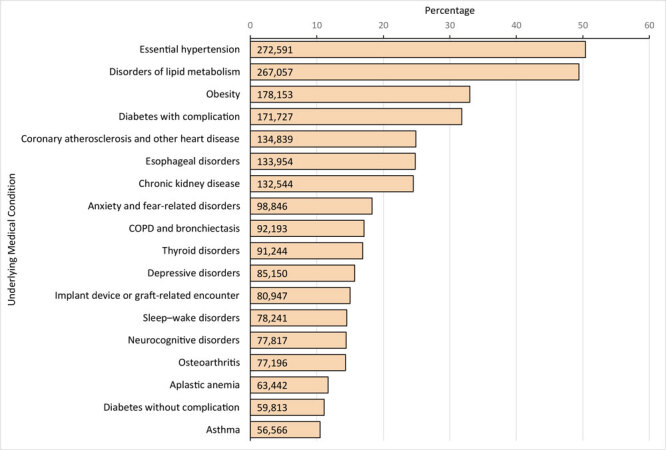
Prevalence of the most frequent underlying medical conditions in a sample of adults hospitalized with COVID-19 in Premier Healthcare Database Special COVID-19 Release. Underlying medical conditions were defined by 1) using Chronic Condition Indicator to identify chronic *International Classification of Diseases, Tenth Revision, Clinical Modification* codes; 2) aggregating the codes into a smaller number of categories by using the Clinical Classifications Software Refined (CCSR); 3) a clinical review of CCSR categories that classified CCSR codes as “likely underlying,” “indeterminate,” or “likely acute”; and 4) including only “likely underlying” CCSR categories and excluding “indeterminate” and “likely acute” CCSR categories. Patients coded with both CCSR categories of “diabetes with complication” and “diabetes without complication” (n = 55,141) were classified as having diabetes with complication. The following frequent (present in ≥10.0% of patients) “indeterminate” CCSR categories were excluded: cardiac dysrhythmias (n = 124,367 [23.0%]), heart failure (n = 104,858 [19.4%]), other specified nervous system disorders (n = 89,929 [16.6%]), other specified and unspecified nutritional and metabolic disorders (n = 89,337 [16.5%]), coagulation and hemorrhagic disorders (n = 75,766 [14.0%]), and diseases of white blood cells (n = 57,765 [10.7%]). Abbreviation: COPD, chronic obstructive pulmonary disease.

We found 18 underlying CCSR categories with a frequency of 10.0% or more in the sample; the most common were essential hypertension (272,591 [50.4%]), disorders of lipid metabolism (267,057 [49.4%]; top ICD-10-CM code was hyperlipidemia), obesity (178,153 [33.0%]), diabetes with complication (171,727 [31.8%]), and coronary atherosclerosis and other heart disease (134,839 [24.9%]) (Figure 1). 

**Table:** 

Underlying Medical Condition	No. (%)
Essential hypertension	272,591 (50.4)
Disorders of lipid metabolism	267,057 (49.4)
Obesity	178,153 (33.0)
Diabetes with complication	171,727 (31.8)
Coronary atherosclerosis and other heart disease	134,839 (24.9)
Esophageal disorders	133,954 (24.8)
Chronic kidney disease	132,544 (24.5)
Anxiety and fear-related disorders	98,846 (18.3)
COPD and bronchiectasis	92,193 (17.1)
Thyroid disorders	91,244 (16.9)
Depressive disorders	85,150 (15.7)
Implant device or graft-related encounter	80,947 (15.0)
Sleep–wake disorders	78,241 (14.5)
Neurocognitive disorders	77,817 (14.4)
Osteoarthritis	77,196 (14.3)
Aplastic anemia	63,442 (11.7)
Diabetes without complication	59,813 (11.1)
Asthma	56,566 (10.5)

Relative risk of death in the full model was 30% higher with obesity (95% CI, 27%–33%), 28% higher with anxiety and fear-related disorders (95% CI, 25%–31%), 26% higher with diabetes with complication (95% CI, 24%–28%), 21% higher with CKD (95% CI, 19%–24%), 18% higher with neurocognitive disorders including dementia and Alzheimer’s disease (95% CI, 15%–21%), 18% higher with chronic obstructive pulmonary disease and bronchiectasis (95% CI, 16%–20%), 17% higher with aplastic anemia including anemia in CKD (95% CI, 14%–19%), 14% higher with coronary atherosclerosis and other heart disease (95% CI, 12%–16%), and 4% higher with thyroid disorders including hypothyroidism (95% CI, 2%–6%) (Table 2). These conditions were also associated with a higher risk of IMV and ICU admission.

**Table 2:** Adjusted Risk Ratios (95% CI) of ICU Admission, IMV, and Death, by Frequent Underlying Medical Conditions^a^ Among Adults Hospitalized With COVID-19 in Premier Healthcare Database Special COVID-19 Release (PHD-SR), March 2020–March 2021

Underlying Medical Condition (CCSR Category)^b^	Death		IMV		ICU Admission	
	Full Model^c^	Restricted Model^d^	Full Model^c^	Restricted Model^d^	Full Model^c^	Restricted Model^d^
**Higher risk of death, IMV, and ICU admission^e^**						
Obesity	1.30 (1.27–1.33)	1.37 (1.34–1.41)	1.50 (1.46–1.54)	1.62 (1.58–1.67)	1.16 (1.14–1.18)	1.20 (1.18–1.23)
Anxiety and fear-related disorders	1.28 (1.25–1.31)	1.29 (1.26–1.32)	1.37 (1.34–1.40)	1.38 (1.34–1.41)	1.14 (1.13–1.16)	1.16 (1.14–1.18)
Diabetes with complication	1.26 (1.24–1.28)	1.44 (1.41–1.46)	1.43 (1.40–1.46)	1.63 (1.59–1.66)	1.16 (1.15–1.18)	1.24 (1.21–1.26)
Chronic kidney disease	1.21 (1.19–1.24)	1.52 (1.49–1.55)	1.08 (1.05–1.11)	1.46 (1.43–1.50)	1.03 (1.02–1.04)	1.18 (1.16–1.20)
Neurocognitive disorders	1.18 (1.15–1.21)	1.19 (1.16–1.21)	1.00 (0.97–1.03)	1.01 (0.98–1.04)	1.04 (1.03–1.06)	1.05 (1.03–1.07)
Chronic obstructive pulmonary disease and bronchiectasis	1.18 (1.16–1.20)	1.27 (1.24–1.29)	1.18 (1.16–1.21)	1.30 (1.27–1.33)	1.09 (1.08–1.10)	1.14 (1.12–1.16)
Aplastic anemia	1.17 (1.14–1.19)	1.46 (1.42–1.49)	1.21 (1.18–1.25)	1.49 (1.45–1.53)	1.11 (1.09–1.12)	1.21 (1.19–1.24)
Coronary atherosclerosis and other heart disease	1.14 (1.12–1.16)	1.28 (1.26–1.30)	1.10 (1.08–1.12)	1.27 (1.25–1.30)	1.08 (1.06–1.09)	1.15 (1.13–1.17)
Thyroid disorders	1.04 (1.02–1.06)	1.10 (1.08–1.12)	1.05 (1.03–1.07)	1.12 (1.09–1.14)	1.04 (1.03–1.05)	1.07 (1.06–1.08)
**Lower risk or nonsignificant difference in the risk of death, IMV, and ICU admission^e^**						
Diabetes without complication	0.94 (0.91–0.97)	0.88 (0.85–0.90)	0.91 (0.88–0.94)	0.91 (0.87–0.94)	0.98 (0.97–0.998)	0.98 (0.97–0.99)
Essential hypertension	0.92 (0.90–0.93)	0.83 (0.81–0.84)	0.94 (0.92–0.95)	0.90 (0.88–0.91)	0.99 (0.97–0.999)	0.97 (0.96–0.99)
Disorders of lipid metabolism	0.94 (0.92–0.95)	1.07 (1.05–1.09)	0.96 (0.94–0.98)	1.13 (1.11–1.16)	0.99 (0.98–1.00)	1.07 (1.05–1.09)
Sleep–wake disorders	0.94 (0.92–0.96)	1.12 (1.10–1.14)	1.00 (0.98–1.03)	1.27 (1.23–1.30)	0.99 (0.98–1.01)	1.10 (1.08–1.12)
Esophageal disorders	0.96 (0.94–0.97)	1.03 (1.02–1.05)	0.95 (0.93–0.96)	1.05 (1.02–1.07)	0.98 (0.97–0.99)	1.03 (1.01–1.05)
Depressive disorders	0.89 (0.87–0.90)	1.04 (1.02–1.06)	0.86 (0.84–0.88)	1.04 (1.02–1.07)	0.96 (0.94–0.97)	1.05 (1.03–1.07)
Osteoarthritis	0.91 (0.90–0.93)	1.00 (0.98–1.02)	0.88 (0.86–0.90)	1.00 (0.97–1.02)	0.95 (0.93–0.97)	1.01 (0.98–1.03)
Implant device or graft related encounter	0.98 (0.97–1.00)	1.09 (1.08–1.11)	0.91 (0.89–0.93)	1.04 (1.02–1.07)	0.98 (0.96–0.99)	1.04 (1.02–1.06)
Asthma	0.91 (0.89–0.94)	0.95 (0.93–0.97)	0.96 (0.94–0.99)	1.03 (1.01–1.06)	1.01 (0.99–1.02)	1.04 (1.02–1.05)
No. of observations	540,667	540,667	506,288	506,288	523,088	523,088

Abbreviations: ICU, intensive care unit; IMV, invasive mechanical ventilation; CCSR, Clinical Classifications Software Refined.

a Underlying medical conditions were defined by 1) using Chronic Condition Indicator to identify chronic *International Classification of Diseases, Tenth Revision, Clinical Modification* codes; 2) aggregating the codes into a smaller number of meaningful categories by using the CCSR; 3) a clinical review of CCSR categories that classified the CCSR codes as “likely underlying,” “indeterminate,” and “likely acute”; and 4) including only “likely underlying” CCSR categories and excluding “indeterminate” and “likely acute” CCSR categories.

b The reference category for each condition is the absence of that condition; the reference category for diabetes with complication and diabetes without complication is the absence of diabetes.

c Full model: Each column includes the results of a single generalized linear model (with Poisson distribution and log link function) that includes all 18 of the most frequent underlying medical conditions (reference: absence of the condition), age group, sex, race/ethnicity, payer type, hospital urbanicity, US Census region of hospital, admission month, and admission month squared.

d Restricted model: Each column includes the results of 18 general linear models (with Poisson distribution and log link function), each including only the underlying medical condition (reference: absence of the condition), age group, sex, race/ethnicity, payer type, hospital urbanicity, US Census region of hospital, admission month, and admission month squared. Patients who died without using ICU care or IMV were excluded from the sample when estimating the model with the outcome of ICU care or IMV, respectively.

e Based on the results from the full model.

Diabetes without complication was associated with a 6% lower risk of death (aRR = 0.94; 95% CI, 0.91–0.97), 9% lower risk of IMV (aRR = 0.91; 95% CI, 0.88–0.94), and 2% lower risk of ICU admission (aRR = 0.98; 95% CI, 0.97–0.998). Essential hypertension was associated with an 8% lower risk of death (aRR = 0.92; 95% CI, 0.90–0.93), 6% lower risk of IMV (aRR = 0.94; 95% CI, 0.92–0.95), and a 1% lower risk of ICU admission (aRR = 0.99; 95% CI, 0.97–0.999). Asthma was associated with a 9% lower risk of death (aRR = 0.91; 95% CI, 0.89–0.94) and a 4% lower risk of IMV (aRR = 0.96; 95% CI, 0.94–0.99).

Age-stratified analysis showed that the number of frequent underlying medical conditions (present in ≥10.0% of patients) was higher with older age (Table 3). The most frequent conditions were obesity, diabetes, and essential hypertension among patients younger than 65, and disorders of lipid metabolism, essential hypertension, diabetes, and coronary atherosclerosis among patients aged 65 or older. Among patients aged 18 to 39, essential hypertension was associated with a 26% higher risk of death (95% CI, 10%–44%), 25% higher risk of IMV (95% CI, 17%–35%), and an 11% higher risk of ICU admission (95% CI, 7%–15%). In the same age group, asthma was frequent and was associated with a 9% (95% CI, 5%–13%) higher risk of ICU admission but was not significantly associated with higher risk of IMV or death. Other specified status (CCSR category indicating a need for specific medical support, such as a wheelchair or renal dialysis) was a frequent category among patients aged 40 to 64 and 65 or older and was associated with a 7% (1%–13%) and 4% (1%–6%) higher risk of death, respectively.

**Table 3:** Most Frequent Underlying Medical Conditions^a^ and Their Association With the Risk of ICU Admission, IMV, and Death Among Adults Hospitalized With COVID-19, Stratified By Age Group, in Premier Healthcare Database Special COVID-19 Release (PHD-SR), March 2020–March 2021

Underlying Medical Condition (CCSR Category)^b^	No. (%)	Risk Ratio (95% CI)^c^		
		Death	IMV	ICU Admission
**Aged 18–39 (n = 59,697)**				
Obesity	22,055 (36.9)	2.20 (1.96–2.47)	1.76 (1.63–1.89)	1.30 (1.26–1.35)
Diabetes	10,648 (17.9)	—	—	—
With complication	7,737 (13.0)	1.84 (1.62–2.08)	1.70 (1.59–1.83)	1.58 (1.51–1.65)
Without complication	2,911 (4.9)	1.11 (0.89–1.37)	0.99 (0.87–1.13)	1.13 (1.07–1.18)
Essential hypertension	9,964 (16.7)	1.26 (1.10–1.44)	1.25 (1.17–1.35)	1.11 (1.07–1.15)
Anxiety and fear-related disorders	9,031 (15.1)	1.26 (1.07–1.48)	1.58 (1.45–1.71)	1.28 (1.24–1.33)
Asthma	8,524 (14.3)	0.95 (0.81–1.10)	0.99 (0.91–1.08)	1.09 (1.05–1.13)
Tobacco-related disorders	7,240 (12.1)	0.81 (0.68–0.98)	0.97 (0.89–1.05)	1.07 (1.03–1.11)
Depressive disorders	5,980 (10.0)	0.96 (0.79–1.17)	0.89 (0.80–0.98)	0.99 (0.94–1.03)
**Aged 40–64 (n = 195,897)**				
Essential hypertension	98,498 (50.3)	0.92 (0.89–0.95)	0.96 (0.94–0.99)	0.99 (0.98–1.00)
Diabetes	87,009 (44.4)	—	—	—
With complication	62,980 (32.1)	1.43 (1.38–1.48)	1.51 (1.46–1.55)	1.18 (1.16–1.21)
Without complication	24,029 (12.3)	0.93 (0.88–0.99)	0.92 (0.88–0.96)	0.97 (0.95–0.99)
Obesity	82,782 (42.3)	1.54 (1.47–1.61)	1.58 (1.52–1.63)	1.16 (1.13–1.19)
Disorders of lipid metabolism	79,899 (40.8)	0.91 (0.88–0.95)	0.95 (0.92–0.98)	0.98 (0.97–1.00)
Esophageal disorders	42,121 (21.5)	0.93 (0.89–0.96)	0.92 (0.90–0.95)	0.97 (0.95–0.99)
Anxiety and fear-related disorders	36,978 (18.9)	1.35 (1.29–1.41)	1.46 (1.41–1.51)	1.14 (1.12–1.16)
Chronic kidney disease	31,911 (16.3)	1.30 (1.23–1.37)	1.09 (1.05–1.13)	1.04 (1.02–1.06)
Sleep–wake disorders	31,499 (16.1)	0.85 (0.81–0.89)	0.97 (0.93–1.00)	0.99 (0.97–1.01)
Coronary atherosclerosis and other heart disease	29,609 (15.1)	1.14 (1.10–1.19)	1.09 (1.06–1.13)	1.09 (1.07–1.11)
Depressive disorders	28,929 (14.8)	0.83 (0.80–0.87)	0.86 (0.83–0.89)	0.95 (0.93–0.97)
Asthma	25,618 (13.1)	0.90 (0.86–0.94)	0.93 (0.90–0.96)	0.99 (0.97–1.01)
Thyroid disorders	24,118 (12.3)	1.03 (0.99–1.08)	1.05 (1.01–1.09)	1.03 (1.01–1.05)
Other specified status	23,773 (12.1)	1.07 (1.01–1.13)	1.01 (0.96–1.06)	1.01 (0.99–1.03)
Tobacco-related disorders	22,893 (11.7)	0.85 (0.81–0.89)	0.90 (0.87–0.94)	0.99 (0.98–1.01)
Chronic obstructive pulmonary disease and bronchiectasis	21,690 (11.1)	1.32 (1.26–1.38)	1.26 (1.22–1.31)	1.09 (1.07–1.11)
Aplastic anemia	19,726 (10.1)	1.32 (1.24–1.40)	1.30 (1.24–1.36)	1.11 (1.09–1.13)
**Aged ≥65 (n = 285,073)**				
Disorders of lipid metabolism	182,267 (63.9)	0.93 (0.91–0.95)	0.95 (0.93–0.97)	0.99 (0.98–1.00)
Essential hypertension	164,129 (57.6)	0.89 (0.87–0.90)	0.86 (0.85–0.88)	0.94 (0.93–0.96)
Diabetes	133,883 (46.9)	—	—	—
With complication	101,010 (35.4)	1.21 (1.19–1.23)	1.36 (1.33–1.40)	1.11 (1.10–1.13)
Without complication	32,873 (11.5)	0.93 (0.90–0.96)	0.89 (0.86–0.93)	0.97 (0.95–0.99)
Coronary atherosclerosis and other heart disease	103,987 (36.5)	1.12 (1.11–1.14)	1.11 (1.08–1.13)	1.08 (1.07–1.09)
Chronic kidney disease	97,802 (34.3)	1.16 (1.13–1.19)	1.04 (1.02–1.07)	1.02 (1.01–1.04)
Esophageal disorders	86,699 (30.4)	0.96 (0.94–0.97)	0.95 (0.93–0.98)	0.98 (0.96–0.99)
Obesity	73,316 (25.7)	1.23 (1.20–1.25)	1.42 (1.38–1.47)	1.13 (1.11–1.15)
Neurocognitive disorders	71,741 (25.2)	1.11 (1.09–1.14)	0.84 (0.81–0.86)	1.00 (0.98–1.01)
Chronic obstructive pulmonary disease and bronchiectasis	69,781 (24.5)	1.14 (1.12–1.16)	1.16 (1.13–1.18)	1.09 (1.07–1.10)
Implant device or graft-related encounter	64,343 (22.6)	0.98 (0.96–0.99)	0.92 (0.90–0.94)	0.98 (0.97–0.99)
Thyroid disorders	63,889 (22.4)	1.03 (1.01–1.05)	1.03 (1.00–1.05)	1.02 (1.01–1.03)
Osteoarthritis	58,125 (20.4)	0.93 (0.91–0.95)	0.91 (0.89–0.94)	0.97 (0.95–0.99)
Anxiety and fear-related disorders	52,837 (18.5)	1.26 (1.23–1.29)	1.27 (1.24–1.30)	1.11 (1.10–1.13)
Depressive disorders	50,241 (17.6)	0.90 (0.88–0.92)	0.87 (0.84–0.89)	0.95 (0.94–0.97)
Other specified status	45,419 (15.9)	1.04 (1.01–1.06)	0.97 (0.94–1.00)	1.00 (0.98–1.02)
Hyperplasia of prostate	44,618 (15.7)	0.91 (0.89–0.93)	0.90 (0.88–0.92)	0.97 (0.96–0.98)
Sleep–wake disorders	43,027 (15.1)	0.98 (0.95–1.00)	1.03 (1.00–1.06)	1.00 (0.98–1.01)
Aplastic anemia	41,091 (14.4)	1.07 (1.05–1.10)	1.09 (1.06–1.13)	1.07 (1.05–1.09)
Malnutrition	32,036 (11.2)	1.39 (1.35–1.43)	1.68 (1.61–1.76)	1.22 (1.19–1.25)
Nervous system pain and pain syndromes	31,129 (10.9)	0.94 (0.91–0.96)	0.89 (0.86–0.91)	0.95 (0.94–0.97)

Abbreviations: ICU, intensive care unit; IMV, invasive mechanical ventilation; CCSR, Clinical Classifications Software Refined.

a Underlying medical conditions were defined by 1) using Chronic Condition Indicator to identify chronic *International Classification of Diseases, Tenth Revision, Clinical Modification* codes; 2) aggregating the codes into a smaller number of meaningful categories by using the CCSR; 3) a clinical review of CCSR categories that classified the CCSR codes as “likely underlying,” “indeterminate,” and “likely acute”; 4) including only “likely underlying” CCSR categories and excluding “indeterminate” and “likely acute” CCSR categories.

b The reference category for each condition is the absence of that condition; the reference category for diabetes with complication and diabetes without complication is the absence of diabetes.

c Each column includes the results of a generalized linear model (with Poisson distribution and log link function), stratified by age group (18–39, 40–64, ≥65) that includes frequent (present in ≥10.0% of patients) underlying medical conditions, age, sex, race/ethnicity, payer type, hospital urbanicity, US Census region of hospital, admission month, and admission month squared. Patients who died without using ICU care or IMV were excluded from the sample when estimating the model with the outcome of ICU care or IMV, respectively.

**Figure 2:**
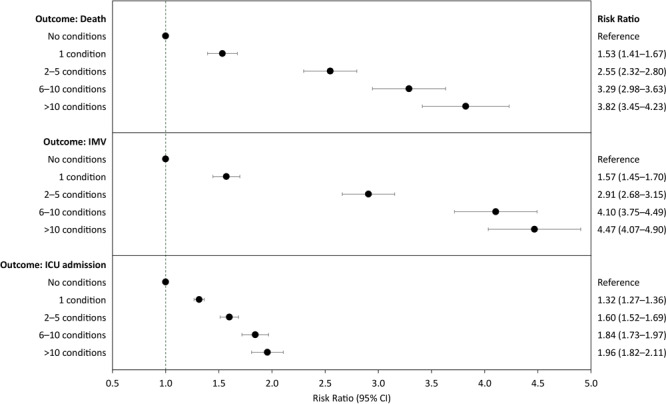
Risk ratio (95% CI) of death, invasive mechanical ventilation (IMV), and admission to intensive care unit (ICU), by the number of underlying medical conditions among adults hospitalized with COVID-19 in the Premier Healthcare Database Special COVID-19 Release. Each panel contains the results of a single generalized linear model with Poisson distribution and log link function, adjusted for age group, sex, race/ethnicity, payer type, hospital urbanicity, US Census region of hospital, admission month, and admission month squared as controls. Patients who died without ICU care or IMV were excluded from the sample when estimating the model with the outcome of ICU care or IMV, respectively.

We found a dose–response association between the total number of underlying medical conditions and risk of severe COVID-19 illness (Figure 2). Compared with patients with no documented underlying medical conditions, patients’ risk of death was 1.53 times (95% CI, 1.41–1.67) as high if they had 1 condition, 2.55 times (95% CI, 2.32–2.80) as high if they had 2 to 5 conditions, 3.29 times (95% CI, 2.98–3.63) as high if they had 6 to 10 conditions, and 3.82 times (95% CI, 3.45–4.23) as high if they had more than 10 conditions. Adjusted RRs for IMV ranged from 1.57 (95% CI, 1.45–1.70) with 1 condition to 4.47 (95% CI, 4.07–4.90) with more than 10 conditions. Adjusted risk ratios for ICU admission ranged from 1.32 (95% CI =1.27–1.36) for patients with 1 condition to 1.96 (95% CI, 1.82–2.11) for patients with more than 10 conditions.

**Table:** 

Outcome	Risk Ratio (95% CI)
**Death**	
No conditions	Reference
1 condition	1.53 (1.41–1.67)
2–5 conditions	2.55 (2.32–2.80)
6–10 conditions	3.29 (2.98–3.63)
>10 conditions	3.82 (3.45–4.23)
**IMV**	
No conditions	Reference
1 condition	1.57 (1.45–1.70)
2-5 conditions	2.91 (2.68–3.15)
6-10 conditions	4.10 (3.75–4.49)
>10 conditions	4.47 (4.07–4.90)
**ICU admission**	
No conditions	Reference
1 condition	1.32 (1.27–1.36)
2-5 conditions	1.60 (1.52–1.69)
6-10 conditions	1.84 (1.73–1.97)
>10 conditions	1.96 (1.82–2.11)

In the first sensitivity analysis, performed by using all CCSR categories, we identified 6 additional frequent “indeterminate” CCSR categories: cardiac dysrhythmias (n = 124,367 [23.0%]), heart failure (n = 104,858 [19.4%]), other specified nervous system disorders (n = 89,929 [16.6%]; top ICD-10-CM code, metabolic encephalopathy), other specified and unspecified nutritional and metabolic disorders (n = 89,337 [16.5%]; top code, hypomagnesemia), coagulation and hemorrhagic disorders (n = 75,766 [14.0%]), and diseases of white blood cells (n = 57,765 [10.7%]). The risk ratio estimates of most previously found underlying conditions were lower with the inclusion of these 6 conditions in the full models.

In the second sensitivity analysis, which used a subset of 278,215 patients with at least 1 encounter in the PHD-SR before their first COVID-19 hospitalization, diabetes without complication was associated with an 8% (95% CI, 5%–12%) higher risk of death, a 13% (95% CI, 10%–17%) higher risk of IMV, and a 5% (95% CI, 4%–7%) higher risk of ICU admission; sleep–wake disorders were associated with an 8% (95% CI, 5%–11%) higher risk of IMV. Anxiety and fear-related disorders were associated with a 2% (95% CI, 0.4%–4%) higher risk of ICU admission but not with a higher risk of death or IMV, on the basis of the full model.

## Discussion

Among 4,899,447 hospitalized US adults in the PHD-SR, 540,667 (11.0%) were hospitalized with COVID-19. Among patients hospitalized with COVID-19, we found 18 most frequent underlying conditions, of which 9 were associated with severe COVID-19 illness. These 9 conditions were both prevalent in the sample (affecting 81.9% of inpatients with COVID-19) and associated with severe COVID-19 illness, suggesting a high impact at the population level. Essential hypertension and disorders of lipid metabolism were the most frequent conditions, whereas obesity, anxiety and fear-related disorders, diabetes with complication, and CKD were the strongest risk factors for death among hospitalized patients with COVID-19. This analysis builds on 2 previous analyses using data from the PHD-SR (3,5), by including more underlying medical conditions in the frequency analysis (274 CCSR categories), including 9 additional months of data, and examining outcomes other than mortality. The analysis also shows that the total number of underlying conditions is strongly associated with severe COVID-19 illness.

The percentage of the US adult population known to have 2 or more underlying medical conditions ranges from approximately 38% to 64% by state (12). Previous studies demonstrated that patients with medically attended COVID-19 often had multiple underlying medical conditions (6). However, studies have rarely focused on the effect of the number of conditions on severe COVID-19 illness. We found that the risk of death, IMV, and ICU admission was often incrementally higher with a higher number of underlying medical conditions. Our finding that the number of underlying medical conditions is itself a risk factor for severe disease from COVID-19 identifies a population that has not been clearly described in previous literature.

Our results reinforce previous findings of higher risk of severe illness associated with diabetes with complication (13), obesity (4,14), coronary atherosclerosis and other heart disease (4), chronic obstructive pulmonary disease (15), and neurocognitive disorders (3,4). Additionally, we identified several conditions for which little data exist regarding risk for severe COVID-19 illness, such as thyroid disorders (including hypothyroidism) and anxiety and fear-related disorders.

Hypertension and disorders of lipid metabolism (the most prevalent conditions), and obesity and diabetes with complication (strong risk factors for death, IMV, and ICU admission) are associated with well-described hormonal and inflammatory pathways, also previously shown to be risk factors for severe COVID-19 illness (16). High baseline prevalence of obesity and diabetes, combined with their association with severe COVID-19 illness, suggest that these 2 conditions could have an outsized impact on the population with COVID-19. Prevention and treatment of these conditions may be an important strategy that could improve national resilience against chronic threats and acute crises. Essential hypertension, for which evidence is mixed on its association with severe COVID-19 illness (1), was shown in our analysis to be the most prevalent condition. It was found to be associated with a higher risk of severe COVID-19 illness only among patients aged 18 to 39 but with a lower risk of severe COVID-19 illness among older patients and in the full sample. This finding supports a possible link with severe COVID-19 illness and identifies essential hypertension as a risk factor, especially among younger patients.

Uncomplicated diabetes was found to be negatively associated with the risk of death and IMV. A positive association with risk of ICU admission was found only among patients aged 18 to 39. A previous study showed that although type 2 diabetes was a risk factor for mortality from severe COVID-19 illness, patients with diabetes and well-controlled blood glucose had lower mortality than those with diabetes and poorly controlled blood glucose (13). Our sensitivity analysis of a subset of patients with pre-COVID encounters identified a higher relative risk of death associated with uncomplicated diabetes present before the first COVID hospitalization. Coding bias (uncomplicated diabetes may be less frequently coded in hospitalizations with severe outcomes) (17) or reverse causality (diabetes complications arising from COVID-19 illness or treatment) (18) could explain this finding.

Anxiety and fear-related disorders were a prevalent condition in our sample; they were also the second highest risk factor for death among the underlying conditions considered in our study. The reasons for this finding are likely multifactorial and may include a reduced ability to prevent infection among patients with anxiety disorders, the immunomodulatory and/or cardiovascular effects of medications used to treat these disorders, or severe COVID-19 illness exacerbating anxiety disorders (19,20). In a subset of patients with pre-COVID encounters in our study, anxiety diagnosed before COVID-19 was not independently associated with death or IMV during COVID-19 hospitalization and, therefore, it is also plausible that anxiety was diagnosed during COVID-19 illness and may be a resulting sequela of COVID-19 (21). Future studies could explore the temporal and causal associations between anxiety disorders and severe COVID-19 illness.

Our finding of a positive association of CKD and coronary atherosclerosis and other heart disease with severe COVID-19 illness has been well described at the epidemiologic level (22). We also found that people with neurocognitive disorders (including dementia and Alzheimer’s disease) were at a higher risk of severe COVID-19 illness, which could be associated with difficulties in access to care and difficulties in following safeguarding procedures (23). Our finding of an association of anemia (specifically, anemia in CKD) with severe COVID-19 illness may be driven by a reduced capacity to respond to acute infections in people with this condition (24).

Asthma diagnosis was present among 10.5% of hospitalized patients with COVID-19 in PHD-SR, which is higher than the 8.0% national prevalence of asthma in 2019 (25). At the same time, we found asthma to be associated with a lower risk of death in the full sample; a positive association with ICU admission was found only among patients younger than 40. This finding supports the mixed evidence on asthma as a risk factor for severe COVID-19 illness (1), although the association between asthma and severe COVID-19 illness could differ by the degree of asthma severity (26).

A sensitivity analysis revealed 6 “indeterminate” conditions (such as coagulation and hemorrhagic disorders, cardiac dysrhythmias, and heart failure) that were both frequent and associated with at least 1 severe COVID-19 illness outcome. Without better information on the temporality of these 6 conditions relative to the COVID-19 illness, we were unable to determine whether these were truly underlying conditions (27,28). Our second sensitivity analysis, restricted to 278,215 patients with encounters that preceded the first COVID-19 encounter, found a positive association of sleep–wake disorders and uncomplicated diabetes with severe COVID-19 illness. Weaker associations of other frequent conditions with COVID-19 illness in this analysis (compared with the main results) could be due to under-ascertainment of certain conditions that resulted from using data only for pre-COVID encounters.

Our study has limitations. First, using ICD-10-CM diagnostic codes to identify COVID-19 cases might result in misclassification, although COVID-19 codes in PHD-SR showed high sensitivity and specificity with SARS-CoV-2 test results (29). Second, ICU risk estimates could be biased if ICU admission reflected factors other than severity of COVID-19, such as anticipation of future severity among health care professionals. Third, because our data were observational, we could not establish causal associations between the underlying conditions and severe COVID-19 illness. Fourth, relying on ICD-10-CM codes to identify underlying medical conditions may have underestimated their prevalence. For example, obesity was diagnosed in 33.0% of the patients, which is possibly an underestimate of this condition, given the national prevalence of 42.4% in 2017–2018 (30) and the prevalence of 50.8% among patients with available height and weight data in PHD-SR (14). Fifth, prior literature shows evidence of both increased documentation (31) and underdiagnosis of certain chronic conditions among patients with more severe illness (17). Sixth, the interrelation of the conditions made it difficult to obtain independent associations, which could explain why certain conditions (disorders of lipid metabolism, sleep–wake disorders, esophageal disorders, and depressive disorders) had a positive association with COVID-19 illness when not adjusted for other conditions and a negative association when adjusted for other conditions. These differences could be explained by 1) confounding in the restricted model, 2) lack of independent effects in the full model, or 3) potential overadjustment in the full model by including variables that were on the causal pathway between the condition of interest and the outcome. Seventh, we were unable to assess the associations of current treatment modalities or medications for underlying medical conditions and severe COVID-19 illness because that information was not available in detail. Finally, including only the most frequent underlying medical conditions in the estimations of risk could have caused us to miss less prevalent risk factors of severity; however, conditions of any frequency were accounted for in the “number of conditions” predictor.

Our study found that 9 of 18 frequent underlying medical conditions among adults hospitalized with COVID-19 were associated with severe illness. Combined with the high prevalence of these conditions (affecting 81.9% of hospitalized patients with COVID-19 in PHD-SR), this finding suggests a potentially high impact at the population level. The highest risk of severe COVID-19 illness was associated with obesity, anxiety and fear-related disorders, diabetes with complication, CKD, and neurocognitive disorders. Among patients younger than 40, essential hypertension was also a risk factor for death. The total number of underlying medical conditions was a strong risk factor of severe COVID-19 illness. Preventing COVID-19 in populations with these conditions and multiple conditions should remain a public health priority, along with targeted mitigation efforts and ensuring high uptake of vaccine, when available, in these people and their close contacts.
